# Prognostic Performance of Sequential Organ Failure Assessment, Acute Physiology and Chronic Health Evaluation III, and Simplified Acute Physiology Score II Scores in Patients with Suspected Infection According to Intensive Care Unit Type

**DOI:** 10.3390/jcm12196402

**Published:** 2023-10-08

**Authors:** Sung-Yeon Hwang, In-Kyu Kim, Daun Jeong, Jong-Eun Park, Gun-Tak Lee, Junsang Yoo, Kihwan Choi, Tae-Gun Shin, Kyuseok Kim

**Affiliations:** 1 Department of Emergency Medicine, Samsung Medical Center, Sungkyunkwan University School of Medicine, Seoul 06351, Republic of Korea; sygood.hwang@samsung.com (S.-Y.H.); jongeun7.park@samsung.com (J.-E.P.);; 2 Department of Digital Health, Samsung Advanced Institute for Health Sciences & Technology, Sungkyunkwan University, Seoul 06351, Republic of Korea; 3 Department of Emergency Medicine, CHA Bundang Medical Center, CHA University School of Medicine, Seongnam 13496, Republic of Korea

**Keywords:** sepsis, mortality, organ dysfunction scores, severity of illness index

## Abstract

We investigated the prognostic performance of scoring systems by the intensive care unit (ICU) type. This was a retrospective observational study using data from the Marketplace for Medical Information in the Intensive Care IV database. The primary outcome was in-hospital mortality. We obtained Sequential Organ Failure Assessment (SOFA), Acute Physiology and Chronic Health Evaluation (APACHE) III, and Simplified Acute Physiology Score (SAPS) II scores in each ICU type. Prognostic performance was evaluated with the area under the receiver operating characteristic curve (AUROC) and was compared among ICU types. A total of 29,618 patients were analyzed, and the in-hospital mortality was 12.4%. The overall prognostic performance of APACHE III was significantly higher than those of SOFA and SAPS II (0.807, [95% confidence interval, 0.799–0.814], 0.785 [0.773–0.797], and 0.795 [0.787–0.811], respectively). The prognostic performance of SOFA, APACHE III, and SAPS II scores was significantly different between ICU types. The AUROC ranges of SOFA, APACHE III, and SAPS II were 0.723–0.826, 0.728–0.860, and 0.759–0.819, respectively. The neurosurgical and surgical ICUs had lower prognostic performance than other ICU types. The prognostic performance of scoring systems in patients with suspected infection is significantly different according to ICU type. APACHE III systems have the highest prediction performance. ICU type may be a significant factor in the prognostication.

## 1. Introduction

In intensive care units (ICUs), it is common to use clinical scoring systems to predict patient outcomes [1]. Predicting patient outcomes helps intensivists in triaging or categorizing critically ill patients, supporting clinical decision-making and allocation of ICU resources. Therefore, modeling the risk of mortality in ICU patients has received considerable attention over the past several decades [2,3,4,5,6,7,8,9,10,11,12,13,14,15,16,17]. Several clinical scoring systems, including Acute Physiology and Chronic Health Evaluation (APACHE), the Simplified Acute Physiology Score (SAPS), and the Mortality Probability Model (MPM), have been developed to predict mortality following ICU admission [4,11,17].

The SOFA score was created by the Working Group of the European Society of Intensive Care Medicine, which aimed to describe as quantitatively and objectively as possible the degree of organ dysfunction/failure in sepsis patients [2]. It was not developed to predict the outcomes of patients. However, because multiorgan dysfunction is the primary cause of mortality in ICU patients, the SOFA score has also been used to predict prognosis in a wide range of critically ill patients, such as those with sepsis, cardiac illness, cardiac surgery, and post-cardiac arrest syndrome [5,7,18,19,20,21,22].

The prognostic performance of critical care scoring systems can vary significantly based on factors such as region, disease, and other variables [23,24,25]. The characteristics and outcomes of patients may be different according to the ICU type [26]. Therefore, the prognostic performance of scoring systems could be different. However, there have been no studies conducted to compare the prognostic performance of these scores across types of ICU. With this background, critical care physicians could not use scoring systems with consideration of ICU types. 

Here, we investigated and compared the prognostic performance of scoring systems in patients with suspected infections in various ICU types using the Marketplace for Medical Information in Intensive Care (MIMIC)-IV database.

## 2. Materials and Methods

### 2.1. Study Setting and Data Source

This retrospective observational study was conducted using data from the MIMIC-IV database, which contain de-identified, comprehensive clinical information for patients admitted from 2008–2019 to the Beth Israel Deaconess Medical Center, a tertiary academic medical center in Boston, Massachusetts, United States. Following successful completion of a data use agreement and “Protection of Human Subjects” training, the MIMIC-IV database can be accessed by the researchers at “https://mimic.physionet.org”. We obtained authorization and accessed the MIMIC-IV database on 27 October 2021. As a result of removal of patient identifiers and de-identification of data information, approval from neither patients nor the ethics committee was required.

### 2.2. Study Population 

We enrolled patients (1) who were ≥18 years old, (2) admitted to the ICU, and (3) who met the criteria for infection suspicion on the first day of ICU admission. The presence of one of the following was considered a cause for infection suspicion: (1) a microbiology culture was performed within the first 24 h after antibiotics were prescribed, and the culture occurred on the first day of ICU admission, or (2) antibiotics were prescribed within the first 72 h after a microbiology culture, and the antibiotic prescription occurred on the first day of ICU admission. In cases where a patient was admitted to the ICU multiple times, only their initial admission was included in the analysis. 

### 2.3. Data Extraction

Researchers who completed the Collaborative Institutional Training Initiative (CIIT) program extracted the following data using the PostgreSQL tools version 10.16 (PostgreSQL Global Development Group, Berkeley, CA, USA) and Python version 3.7: age, sex, comorbidities, Charlson comorbidity score [27,28], type of ICU, vital signs, first-day laboratory results, Glasgow coma scale, vasoactive drugs, organ support interventions such as mechanical ventilation and renal replacement therapy, and in-hospital mortality rates. The data codes for the study analyses were from the MIMIC Code Repository [29]. Only the first suspected infection episode per encounter was identified. The ICU types were cardiac care unit (CCU), cardiovascular ICU (CVICU), medical ICU (MICU), neurosurgical ICU (NSICU), neuroscience ICU (neuro-ICU), surgical ICU (SICU), mixed unit (MICU and SICU), and trauma SICU. 

### 2.4. Calculation of SOFA, APACHE III, and SAPS II Scores

The component of the SOFA score was determined using the worst available measurement for each variable from 6 h prior to ICU admission to 24 h after. In accordance with the methodology of prior studies, we assigned a score of 0 points to any SOFA variables that were absent from our analysis and assumed that they were normal [30]. Only vasoactive medications (dopamine, epinephrine, norepinephrine, and dobutamine) used for >1 h consecutively between 6 h prior to ICU admission to 24 h after ICU admission were used to calculate the cardiovascular SOFA subscore. If multiple medications were used for >1 h, the drug with the highest SOFA score was analyzed. Using the PaO_2_/FiO_2_ ratio calculated from 6 h prior to ICU admission to 24 h after, the respiratory SOFA subscore was determined. 

The APACHE III score was calculated for each patient from data collected during the first 24 h of ICU admission. Knaus et al. first proposed using the APACHE III scoring system to evaluate patients with severe diseases in 1991 [9]. This score is composed of an acute physiology score, chronic health status score, and age score. 

The SAPS II score was also extracted from the data on the first day of each ICU patient’s stay. The SAPS II score was proposed in 1993, using a larger sample from 12 countries, and it includes 12 physiologic variables, age, type of admission, and underlying disease variables [6]. We used the worst value of each variable during the first 24 h of ICU admission, and missing values were considered normal for APACHE III and SAPS II score calculation. 

### 2.5. Outcome

The primary outcome was in-hospital mortality. There is no secondary outcome. 

### 2.6. Statistical Analysis

Categorical data are presented as frequencies and percentages, whereas continuous data are described as mean (standard deviation). Student’s *t* test was used to compare continuous data, and the chi-square test was used to evaluate categorical variables. The discrimination and calibration of the total SOFA score and each component of the SOFA score for predicting in-hospital mortality according to ICU type were assessed utilizing the area under the receiver operating characteristic (AUROC) and calibration curves, respectively. Likewise, the discriminating performance of the APACHE III and SAPS II scores in predicting in-hospital mortality was evaluated using the AUROC. Youden’s index was calculated using the ROC curve and coordinates to determine the optimal cutoff values for SOFA, APHACE III, and SAPS II scores. A cutoff value was chosen based on the highest Youden index, and the sensitivity, specificity, positive predictive value, and negative predictive value were calculated using Youden’s index. *p* < 0.05 was considered statistically significant. R version 3.6.3 (R Foundation for Statistical Computing, Vienna, Austria) and STATA version 17.0 (StataCorp LLC, College Station, TX, USA) were used for statistical analysis.

## 3. Results

### 3.1. Baseline Characteristics

**Figure 1:**
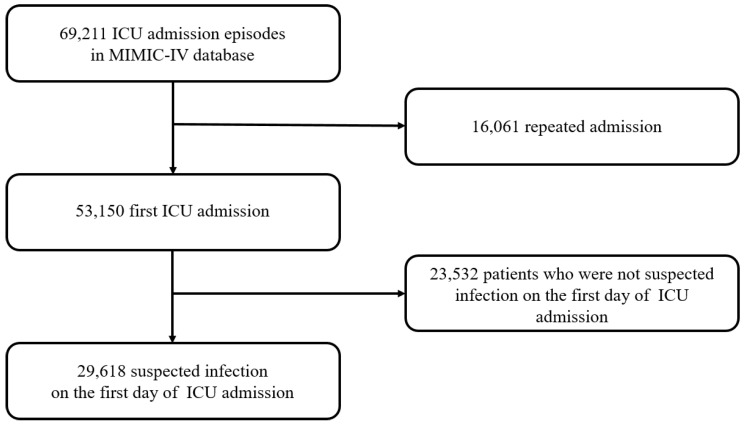
Flowchart of the study population.

A flowchart illustrating the research process is presented in Figure 1. During the study period, we screened a total of 69,211 ICU admission episodes. After excluding 16,061 repeated admission episodes and 23,532 patients who met the exclusion criteria, 29,618 patients were analyzed, including 3675 (12.4%) who died in the hospital. Table 1 shows the baseline characteristics of the study population. The mean age of the study population was 65.4 ± 16.7 years, and 56.7% of the patients were male. The proportion of patients according to ICU type was 22.8% in the CVICU, 22.1% in the MICU, 19.5% in the mixed unit (MICU and SICU), and 13.4% in the SICU. There was a significant difference in ICU type between survivors and non-survivors (*p* < 0.001). The mean SOFA, APACHE III, and SAPS II scores were 5.5 ± 3.8, 61.1 ± 24.6, and 37.0 ± 14.5 points, respectively.

### 3.2. In-Hospital Mortality and ICU Scores According to ICU Type

In-hospital mortality and the three scores according to the type of ICU are presented in Table 2. The in-hospital mortality rate was highest in the NSICU (17.5%), followed by in the CCU, MICU, mixed unit, and SICU. On the other hand, the MICU had the highest mean SOFA score and APACHE III score, while the SAPS II score was highest in the mixed unit and CCU. The mean scores of the six SOFA components according to type of ICU are shown in Table S1. 

### 3.3. SOFA Score According to ICU Type

The AUROC of the total SOFA score to predict in-hospital mortality for overall patients was 0.785 (95% confidence interval [CI], 0.773–0.797) (Table 3). AUROCs were significantly different according to ICU type (*p* < 0.001). The CCU had the highest AUROC (0.826). Compared to the MICU (0.793), the NSICU (0.723) and the SICU (0.748) had significantly lower AUROCs. Discrimination of the six components of the SOFA score was also heterogeneous, and the AUROC of the coagulation SOFA subscore was relatively low (AUROC, 0.566) (Table S2). 

The cutoff points, sensitivities, specificities, positive predictive value (PPV), and negative predictive value (NPV) of the SOFA score according to ICU type are presented in Table S3. The optimal cutoff of SOFA score for predicting in-hospital mortality was 6.5 based on Youden’s index, with an AUROC of 0.721, a sensitivity of 71.2%, a specificity of 72.9%, a PPV of 27.2%, and an NPV of 94.7%. The cutoff points ranged from 3.5–7.5, and AUROCs ranged from 0.678–0.759. Other statistics also varied according to ICU type. 

The calibration of the SOFA score for in-hospital mortality was evaluated using a calibration curve that compared the expected probability and the observed probability (Figure S1). In all patients, calibration was fair in the total SOFA score and subscores of the six components. However, the calibration curve varied greatly by ICU type, with good calibration in the MICU and SICU but poor calibration in the CVICU, NSICU, and neuro-ICU. In the six components of SOFA, overall calibration for all patients was good except for the cardiovascular and coagulation SOFA subscores, and calibration was heterogeneous according to ICU type. 

### 3.4. APACHE III Score According to ICU Type

The AUROC of the APACHE III score for in-hospital mortality was highest among the three scoring systems (0.807; 95% CI, 0.799–0.814) (Table 3). AUROCs were significantly different according to ICU type (*p* < 0.001). The neuro-ICU had the highest AUROC (0.860), followed by the CVICU (0.837). Compared to the MICU (0.796), the NSICU (0.728) and the SICU (0.766) had significantly lower AUROCs.

The optimal cutoff point was 67.5, with an AUROC of 0.733, a sensitivity of 75.0%, a specificity of 71.7%, a PPV of 27.3%, and an NPV of 95.3% (Table S4). The cutoff ranged from 55.5–79.5, and AUROCs ranged from 0.668–0.812 according to ICU type. Calibration in MICU, SICU, and trauma SICU was good, but calibration in the CVICU, NSICU, and neuro-ICU was relatively poor (Figure S2). 

### 3.5. SAPS II Score According to ICU Type

The AUROC of the SAPS II score to predict in-hospital mortality for overall patients was 0.795 (95% CI, 0.787–0.811) (Table 3). AUROCs were significantly different according to ICU type (*p* = 0.009). The trauma SICU had the highest AUROC (0.819), which was significantly higher than those of the SOFA and APACHE III scores. Compared to the MICU (0.795), the SICU (0.759) had a significantly lower AUROC.

The optimal cutoff point was 41.5, with an AUROC of 0.719, a sensitivity of 70.3%, a specificity of 73.4%, a PPV of 27.3%, and an NPV of 94.6% (Table S5). The cutoff ranged from 36.5–44.5, and AUROCs ranged from 0.687–0.740 according to ICU type. Calibration in the SICU and overall study population was good, but calibration in the CVICU, NSICU, and neuro-ICU was relatively poor (Figure S3). 

## 4. Discussion

This study demonstrates that prognostic scoring systems, such as APACHE III, SAPS II, and SOFA, have heterogeneous performance in terms of discrimination and calibration among ICU types. 

Accurate prognostication in ICU patients is essential for treatment planning, overall improvements in patient care, and risk adjustment of the study population. There are well-known prediction models for critically ill patients, but their accuracy has been challenged [23,24]. Regional variations, disease/time sensitivity, and adherence to current guidelines have been proposed as causes for the wide range of predictive performance. Some studies proposed that scoring systems should be developed and validated in the country that would be using them and would be periodically updated [23]. However, this might be unrealistic, so another option could be to make adjustments for those variable factors. With the results of this study, we might propose that ICU type is also an important adjustment factor for prognostication. 

Currently, clinicians may use internet-based calculation systems such as APACHE III and SAPS II, and, if variables are filled up, the point and in-hospital mortality are demonstrated. However, there is no consideration of ICU types. We showed that the prognostic performance varies considerably among ICU types. Therefore, the mortality prediction might be much improved if the scoring systems have a component for ICU types, hence the importance of this study.

Our study showed that the APACHE III score has significantly greater prognostic performance than either the SOFA or SAPS II score. The performance range of these scoring systems is wide, and the results of comparing the scoring systems are not consistent [24]. This finding also supports the precautious use of scoring systems. 

Scores developed in general populations may have less prognostic accuracy in specific groups, so it is an important issue to reflect differences [16]. In this study, the discrimination power of all three scoring systems was lower in the NSICU and SICU than other ICU types. The investigation of the cause of these results is beyond the scope of this study, but performance of surgery could be one factor. SAPS and the MPM have a component for surgery, but they emphasize emergent surgery [4,11]. In APACHE III, the risk-estimate equation for in-hospital mortality incorporates operative admission and prior patient location [9]. 

The prognostic performance of coagulation SOFA was lowest in this study compared to other system SOFA subscores. As such, it might need modification. One explanation could be that platelet transfusions were not recorded, and this could influence the coagulation SOFA subscore [21]. 

NSICU patients had the lowest CNS SOFA subscores. It is paradoxical that CNS SOFA, reflecting the neurological status of patients, was lowest in the neurosurgical ICU. However, the CNS component in the SOFA score is known to be the least accurately measured and is associated with the most errors [21]. Intubation, sedation, and operation may have negative effects on the accuracy of CNS SOFA [31]. 

Both SAPS III and the MPM have been widely used to predict mortality following ICU admission, and their accuracy is comparable or superior to that of APACHE III or SOFA [3,23,32,33]. It would be interesting to investigate the predictive performance of SAPS III or the MPM according to ICU type. 

In this study, we included suspected infection patients, including sepsis and septic shock, on the first day of ICU admission. In the sepsis diagnosis and management, early, accurate and individualized risk assessment is critical [34,35]. The ICU scoring system is a main tool for this purpose, and consideration of the ICU type as well as sepsis management information may enhance the prognostic performance.

Our study focused on widely used, rule-based models in intensive care for future modification and development. Machine-learning-based prediction models have been developed in emergency and critical care settings and can be considered among the solutions for improving the prognostic performance across ICU types, although there are still barriers for adopting machine-learning models in clinical practice [36,37]. 

This study has several limitations that should be considered. First, this was a retrospective observational study, and there was the possibility of selection bias because the population investigated in this study consisted of patients with suspected infection from a single center in the United States. Also, we used the previous definition using microbial culture and antibiotic uses in the predefined time frame, but this may have led to loss of certain patients with or without infection in ICUs. Therefore, the results should be interpreted with caution when extrapolating to other populations and regions. Second, the numbers of patients in some ICU types were small, which might lead to inaccurate estimation of the prognostic performance in the ICU. Fourth, we did not use the most recent version of ICU scoring systems, and further evaluation will be required. Fifth, due to inaccurate charting of real-time events, this database may contain incomplete or inaccurate information. Lastly, since the scores were mostly obtained directly from pre-existing programming queries, missing data during the calculation process may create variations from the true values. 

## 5. Conclusions

The prognostic performance of scoring systems in patients with suspected infection is significantly different according to ICU type. APACHE III systems have the highest prediction performance. ICU type may be a significant factor in the prognostication. Further large-scale study should be performed and consequently, the ICU type might be included the scoring systems.

## Figures and Tables

**Table 1:** Baseline characteristics of the study population.

Variables	All Patients (n = 29,618)	Survivors (n = 25,943)	In-Hospital Death (n = 3675)	*p*
Age (mean ± SD), years	65.4 ± 16.7	64.7 ± 16.7	69.8 ± 15.6	<0.001
Male sex, No. (%)	16,806 (56.7)	14,833 (57.2)	1973 (53.7)	<0.001
Charlson comorbidity score (mean ± SD)	5.5 ± 2.9	5.3 ± 2.9	7.0 ± 3.0	<0.001
Intensive care unit				<0.001
MICU	6532 (22.1)	5393 (20.8)	1139 (31.0)	
MICU/SICU (mixed)	5787 (19.5)	4836 (18.6)	951 (25.9)	
CCU	2590 (8.7)	2138 (8.2)	452 (12.3)	
CVICU	6747 (22.8)	6562 (25.3)	185 (5.0)	
NSICU	411 (1.4)	338 (1.3)	73 (2.0)	
Neuro-ICU	310 (1.1)	294 (1.1)	16 (0.4)	
SICU	3967 (13.4)	3473(13.4)	494 (13.4)	
Trauma SICU	3274 (11.1)	2909 (11.2)	365 (9.9)	
Vital signs ^a^				
Lowest MAP (mean ± SD), mmHg	61.5 ± 11.5	62.3 ± 11.0	55.8 ± 13.6	<0.001
Highest HR (mean ± SD), beat per min	105.0 ± 20.6	103.9 ± 19.9	113.2 ± 23.4	<0.001
Highest RR (mean ± SD), breath per min	28.4 ± 6.6	28.1 ± 6.5	31.0 ± 7.2	<0.001
Highest temperature, °C	37.4 ± 0.8	37.5 ± 0.8	37.3 ± 1.1	<0.001
Laboratory findings ^a^ (mean ± SD)				
Highest WBCs, 10^9^/L	15.3 ± 11.9	14.9 ± 10.8	18.1 ± 17.4	<0.001
Lowest Hb, g/dL	9.6 ± 2.4	9.6 ± 2.3	9.9 ± 2.7	<0.001
Lowest platelets, 10^9^/L	171.8 ± 81.3	174.0 ± 79.2	155.8 ± 93.0	<0.001
Highest bilirubin, mg/dL	2.1 ± 4.7	1.8 ± 3.8	3.7 ± 7.4	<0.001
Highest creatinine, mg/dL	1.5 ± 1.5	1.4 ± 1.5	2.1 ± 1.7	<0.001
Highest lactate, mmol/L	2.9 ± 2.5	2.6 ± 1.8	4.9 ±4.4	<0.001
GCS score ^a^ (mean ± SD)	13.5 ± 2.9	13.6 ± 2.8	12.8 ± 3.6	<0.001
Vasopressor use ^a^, No. (%)	10,156 (34.3)	8151 (31.4)	2005 (54.6)	<0.001
Norepinephrine, No. (%)	5392 (18.2)	3779 (14.6)	1613 (43.9)	<0.001
Epinephrine, No. (%)	1489 (5.0)	1186 (4.6)	303 (8.2)	<0.001
Dopamine, No. (%)	601 (2.0)	368 (1.4)	233 (6.3)	<0.001
Vasopressin, No. (%)	1611 (5.4)	845 (3.3)	766 (20.8)	<0.001
Phenylephrine, No. (%)	5294 (17.9)	4505 (17.4)	789 (21.5)	<0.001
Dobutamine use, No. (%)	276 (0.9)	165 (0.6)	111 (3.0)	<0.001
Norepinephrine-equivalent dose ^b^ (mean ± SD), µg/kg/min	0.20 ± 0.25	0.15 ± 0.19	0.40 ± 0.37	<0.001
Mechanical ventilation ^a^, No. (%)	14,812 (50.0)	12,590 (48.5)	2222 (60.5)	<0.001
Renal replacement therapy ^a^, No. (%)	629 (2.2)	420 (1.6)	209 (5.7)	<0.001
SOFA score ^a^ (mean ± SD)	5.5 ± 3.8	5.0 ± 3.4	9.0 ± 4.5	<0.001
APACHE III score	61.1 ± 24.6	57.3 ± 21.5	87.7 ± 28.4	<0.001
SAPS II score	37.0 ± 14.5	34.9 ± 12.9	51.8 ± 16.5	<0.001

Data are presented as mean with standard deviation or frequency (%). ^a^ The worst value and intervention on the first day of intensive care unit admission were extracted. ^b^ Norepinephrine-equivalent doses are given as µg/kg/min at least 1 h. APACHE, Acute Physiology and Chronic Health Evaluation; CCU, cardiac care unit; CVICU, cardiovascular intensive care unit; Hb, hemoglobin; HR, heart rate; MAP, mean arterial pressure; MICU, medical intensive care unit; MICU/SICU, medical intensive care unit/surgical intensive care unit; neuro-ICU, neurological intensive care unit; NSICU, neurosurgical intensive care unit; RR, respiratory rate; SAPS, Simplified Acute Physiology Score; SD, standard deviation; SICU, surgical intensive care unit; SOFA, Sequential Organ Failure Assessment; WBCs, white blood cells.

**Table 2:** In-hospital mortality rate and SOFA, APACHE III, and SAPS II scores according to ICU type.

ICU Type	In-Hospital Mortality, %	Total SOFA	APACHE III Score	SAPS II Score
MICU	17.4	6.3 ± 4.4	67.4 ± 25.8	37.9 ± 15.2
MICU/SICU	16.4	5.5 ± 3.9 ^a^	65.7 ± 25.7 ^a^	38.7 ± 15.8 ^a^
CCU	17.5	5.5 ± 4.0 ^a^	65.2 ± 23.7 ^a^	38.7 ± 14.7 ^a^
CVICU	2.7 ^a^	5.6 ± 3.0 ^a^	54.6 ± 20.1 ^a^	36.9 ± 12.5 ^a^
NSICU	17.8	4.8 ± 3.0 ^a^	54.0 ± 20.2 ^a^	35.7 ± 11.9 ^a^
Neuro-ICU	5.2 ^a^	2.8 ± 2.1 ^a^	48.3 ± 18.5 ^a^	31.6 ± 10.9 ^a^
SICU	12.5 ^a^	5.0 ± 3.8 ^a^	58.3 ± 24.3 ^a^	35.3 ± 14.3 ^a^
Trauma SICU	11.2 ^a^	4.8 ± 3.6 ^a^	54.2 ± 23.7 ^a^	33.6 ± 14.2 ^a^

Data are presented as mean with standard deviation or frequency (%). ^a^ *p* < 0.05 compared to the MICU. APACHE, Acute Physiology and Chronic Health Evaluation; CCU, cardiac care unit; CVICU, cardiovascular intensive care unit; ICU, intensive care unit; MICU, medical intensive care unit; MICU/SICU, medical intensive care unit/surgical intensive care unit; neuro-ICU, neurological intensive care unit; NSICU, neurosurgical intensive care unit; SAPS, Simplified Acute Physiology Score; SICU, surgical intensive care unit SOFA, Sequential Organ Failure Assessment.

**Table 3:** AUROC of SOFA, APACHE III, and SAPS II scores for in-hospital mortality according to ICU type.

ICU Type	SOFA Score (95% CI)	APACHE III Score (95% CI)	SAPS II Score (95% CI)
Overall	0.785 (0.773–0.797) ^a,c^	0.807 (0.799–0.814) ^b,c^	0.795 (0.787–0.811) ^a,b^
MICU	0.793 (0.779–0.808)	0.796 (0.782–0.810)	0.795 (0.780–0.809)
MICU/SICU	0.793 (0.778–0.808)	0.801 (0.786–0.816)	0.806 (0.791–0.821)
CCU	0.826 (0.806–0.846) ^d^	0.808 (0.785–0.830)	0.810 (0.788–0.832)
CVICU	0.805 (0.768–0.841)	0.837 (0.812–0.863) ^c,d^	0.799 (0.769–0.830) ^a^
NSICU	0.723 (0.657–0.790) ^d^	0.728 (0.668–0.787) ^d^	0.760 (0.706–0.813)
Neuro-ICU	0.821 (0.732–0.91)	0.860 (0.790–0.930) ^c^	0.803 (0.705–0.902) ^a^
SICU	0.748 (0.725–0.771) ^d^	0.766 (0.742–0.789) ^d^	0.759 (0.737–0.782) ^d^
Trauma SICU	0.786 (0.760–0.812) ^c^	0.795 (0.769–0.819) ^c^	0.819 (0.796–0.841) ^a,b^

^a^ *p* < 0.05 compared to the APACHE III score. ^b^ *p* < 0.05 compared to the SOFA score. ^c^ *p* < 0.05 compared to the SAPS II score. ^d^ *p* < 0.05 compared to the MICU. APACHE, Acute Physiology and Chronic Health Evaluation; AUROC, area under the receiver operating characteristic curve; CCU, cardiac care unit; CI, confidence interval; CVICU, cardiovascular intensive care unit; ICU, intensive care unit; MICU, medical intensive care unit; MICU/SICU, medical intensive care unit/surgical intensive care unit; neuro-ICU, neurological intensive care unit; NSICU, neurosurgical intensive care unit; SAPS, Simplified Acute Physiology Score; SICU, surgical intensive care unit; SOFA, Sequential Organ Failure Assessment.

## Data Availability

The datasets generated and/or analyzed during the current study are available from the corresponding author on reasonable request.

## Supplementary Materials

The following supporting information can be downloaded at: https://www.mdpi.com/article/10.3390/jcm12196402/s1. Table S1. Six components of SOFA according to ICU type; Table S2. AUROC of the six components of SOFA score according to type of intensive care unit; Table S3. SOFA score cutoffs for in-hospital mortality according to ICU type; Table S4. APACHE III score cutoffs for in-hospital mortality based on ICU type; Table S5. SAPS II score cutoffs for in-hospital mortality based on ICU type; Figure S1. Calibration plots of SOFA scores according to ICU type; Figure S2. Calibration plots of APACHE III scores according to ICU type; Figure S3. Calibration plots of SAPS II scores according to ICU type.

## Abbreviations

ICU: intensive care unit; APACHE, Acute Physiology and Chronic Health Evaluation; SAPS, Simplified Acute Physiology Score; MPM, Mortality Probability Model; MIMIC, Marketplace for Medical Information in Intensive Care; CCU, cardiac care unit; CVICU, cardiovascular intensive care unit; MICU medical intensive care unit; NSICU, neurosurgical intensive care unit; neuro-ICU, neuroscience intensive care unit; SICU, surgical intensive care unit; AUROC, area under the receiver operating characteristic; CI, confidence interval.
