# Supplementary material for: Prognostic Performance of Sequential Organ Failure Assessment, Acute Physiology and Chronic Health Evaluation III, and Simplified Acute Physiology Score II Scores in Patients with Suspected Infection According to Intensive Care Unit Type
Source: J Clin Med. 2023 Oct 8;12(19):6402. doi: 10.3390/jcm12196402 (PMC10573563; doi:10.3390/jcm12196402)

Table S1. Six components of SOFA according to ICU type.

ICU type	Cardiovascular SOFA	CNS SOFA	Respiratory SOFA	Renal SOFA	Hepatic SOFA	Coagulation SOFA
MICU	1.5 ± 1.4	1.2 ± 1.3	1.1 ± 1.5	1.3 ± 1.4	0.6 ± 1.1	0.7 ± 1.0
MICU/SICU	1.4 ± 1.3[a]	1.1 ± 1.2[a]	0.8 ± 1.3[a]	1.0 ± 1.3[a]	0.5 ± 1.0[a]	0.7 ± 1.1
CCU	1.6 ± 1.4[a]	1.1 ± 1.3[a]	1.0 ± 1.5	1.2 ± 1.3	0.2 ± 0.7[a]	0.5 ± 0.7[a]
CVICU	1.5 ± 1.0	1.1 ± 1.3[a]	1.7 ± 1.3[a]	0.4 ± 0.8[a]	0.1 ± 0.3[a]	0.8 ± 0.7[a]
NSICU	1.0 ± 1.1[a]	1.8 ± 1.3[a]	0.7 ± 1.2[a]	0.6 ± 1.0[a]	0.2 ± 0.6[a]	0.5 ± 0.8[a]
Neuro-ICU	0.4 ± 0.6[a]	1.3 ± 1.1	0.04 ± 0.3	0.7 ± 1.3[a]	0.04 ± 0.2[a]	0.2 ± 0.5[a]
SICU	1.1 ± 1.2[a]	1.3 ± 1.3[a]	0.8 ± 1.3[a]	0.8 ± 1.2[a]	0.5 ±1.0[a]	0.5 ± 0.9[a]
Trauma SICU	1.2 ± 1.2[a]	1.2 ± 1.2	0.9 ± 1.3[a]	0.7 ± 1.1[a]	0.2 ± 0.7[a]	0.5 ± 0.8[a]

Data are presented as mean with standard deviation and frequency (%).

[a]$P < 0.05$ compared to the MICU.

CCU, cardiac care unit; CNS, central nervous system; CVICU, cardiovascular intensive care unit; ICU, intensive care unit; MICU, medical intensive care unit; MICU/SICU, medical intensive care unit/surgical intensive care unit; neuro-ICU, neurological intensive care unit; NSICU, neurosurgical intensive care unit; SICU, surgical intensive care unit; SOFA, Sequential Organ Failure Assessment.

Table S2. AUROC of the six components of SOFA score according to type of intensive care unit.

ICU type	Cardiovascular SOFA[a] (95% CI)	Coagulation SOFA[a] (95% CI)	Respiration SOFA[a] (95% CI)	Hepatic SOFA[a] (95% CI)	Central nervous system SOFA[a] (95% CI)	Renal SOFA[a] (95% CI)
Overall	0.663 (0.654–0.673)	0.566 (0.556–0.576)	0.619 (0.610–0.629)	0.606 (0.598–0.614)	0.653 (0.642–0.663)	0.701 (0.692–0.710)
MICU	0.687 (0.670–0.703)	0.601 (0.584–0.618)	0.669 (0.652–0.686)	0.624 (0.607–0.640)	0.640 (0.62–0.659)	0.680 (0.660–0.694)
MICU/ SICU	0.648 (0.63–0.666)[b]	0.591 (0.572–0.61)	0.646 (0.628–0.664)	0.549 (0.532–0.566)	0.690 (0.671–0.710)[b]	0.664 (0.642–0.680)
CCU	0.700 (0.673–0.727)	0.538 (0.512–0.564)[b]	0.687 (0.66–0.713)	0.584 (0.561–0.606)[b]	0.707 (0.678–0.735)[b]	0.694 (0.665–0.719)
CVICU	0.708 (0.668–0.749)	0.570 (0.522–0.618)	0.594 (0.545–0.642)[b]	0.617 (0.585–0.65)	0.601 (0.556–0.646)	0.796 (0.763–0.833)[b]
NSICU	0.588 (0.520–0.656)[b]	0.595 (0.529–0.661)	0.572 (0.509–0.635)[b]	0.565 (0.512–0.618)[b]	0.560 (0.469–0.65)	0.663 (0.606–0.737)
Neuro-ICU	0.516 (0.373–0.660)[b]	0.536 (0.427–0.644)	0.558 (0.473–0.642)[b]	0.578 (0.479–0.677)	0.769 (0.652–0.885)[b]	0.629 (0.529–0.779)
SICU	0.630 (0.605–0.656)[b]	0.578 (0.554–0.603)	0.610 (0.585–0.634)[b]	0.582 (0.559–0.605)[b]	0.667 (0.638–0.696)	0.644 (0.619–0.67)[b]
Trauma SICU	0.706 (0.679–0.734)	0.605 (0.576–0.633)	0.655 (0.625–0.685)	0.584 (0.560–0.608)[b]	0.589 (0.553–0.624)[b]	0.696 (0.659–0.718)

[a]*P* < 0.05 by comparison of ICU types.

[b]*P* < 0.05 compared to the MICU.

AUROC, area under the receiver operating characteristic curve; CCU, cardiac care unit; CI, confidence interval; CVICU, cardiovascular intensive care unit; ICU, intensive care unit; MICU, medical intensive care unit; MICU/SICU, medical intensive care unit/surgical intensive care unit; neuro-ICU, neurological intensive care unit; NSICU, neurosurgical intensive care unit; SICU, surgical intensive care unit; SOFA, Sequential Organ Failure Assessment.

Table S3. SOFA score cutoffs for in-hospital mortality according to ICU type.

ICU type	Cutoff	AUROC (95% CI)	Sensitivity (95% CI)	Specificity (95% CI)	Positive predictive value (95% CI)	Negative predictive value (95% CI)
Overall	6.5	0.721 (0.713–0.729)	71.2 (69.7–72.7)	72.9 (72.4–73.5)	27.2 (26.3–28.1)	94.7 (94.4–95.0)
MICU	7.5	0.729 (0.715–0.744)	70.7 (67.9–73.3)	75.2 (74.0–76.3)	37.5 (35.5–39.6)	92.4 (91.6–93.2)
MICU/SICU	6.5	0.725 (0.710–.741)	70.6 (67.5–73.4)	74.5 (73.3–75.7)	35.2 (33.1–37.4)	92.8 (91.9–93.6)
CCU	6.5	0.755 (0.733–0.777)	76.3 (72.1–80.2)	74.6 (72.7–76.5)	38.9 (35.7–42.2)	93.7 (92.5–94.8)
CVICU	7.5	0.759 (0.727–0.792)	73.0 (66.0–79.2)	78.9 (77.9–79.9)	8.9 (7.5–10.4)	99.0 (98.7–99.3)
NSICU	4.5	0.675 (0.619–0.732)	75.3 (63.9–84.7)	59.8 (54.3–65.0)	28.8 (22.5–35.8)	91.8 (87.4–95.1)
Neuro-ICU	3.5	0.757 (0.654–0.859)	81.3 (54.4–96.0)	70.1 (64.5–75.2)	12.9 (7.04–21.0)	98.6 (95.9–99.7)
SICU	4.5	0.689 (0.670–0.709)	79.1 (75.3–82.6)	58.7 (57.1–60.4)	21.4 (19.6– 23.4)	95.2 (94.2–96.1)
Trauma SICU	6.5	0.722 (0.697–0.748)	64.7 (59.5–69.6)	79.8 (78.3–81.2)	28.6 (25.6–31.9)	94.7 (93.8–95.6)

AUROC, area under the receiver operating characteristic curve; CCU, cardiac care unit; CI, confidence interval; CVICU, cardiovascular intensive care unit; ICU, intensive care unit; MICU, medical intensive care unit; MICU/SICU, medical intensive care unit/surgical intensive care unit; neuro-ICU, neurological intensive care unit; NSICU, neurosurgical intensive care unit; SICU, surgical intensive care unit; SOFA, Sequential Organ Failure Assessment.

Table S4. APACHE III score cutoffs for in-hospital mortality based on ICU type.

ICU type	Cutoff	AUROC (95% CI)	Sensitivity (95% CI)	Specificity (95% CI)	Positive predictive value (95% CI)	Negative predictive value (95% CI)
Overall	67.5	0.733 (0.726–0.741)	75.0 (73.6–76.4)	71.7 (71.1–72.2)	27.3 (26.4–28.2)	95.3 (95.0–95.6)
MICU	79.5	0.721 (0.706–0.736)	63.7 (60.9–66.5)	80.4 (79.3–81.4)	40.7 (38.4–43.0)	91.3 (90.5–92.1)
MICU/SICU	74.5	0.729 (0.713–0.745)	69.8 (66.8–72.7)	76.0 (74.7–77.2)	36.3 (34.1–38.6)	92.8 (91.9–93.5)
CCU	69.5	0.741 (0.719–0.763)	77.4 (73.3–81.2)	70.8 (68.8–72.7)	35.9 (32.9–39.0)	93.7 (92.4–94.8)
CVICU	61.5	0.781 (0.753–0.809)	82.7 (76.5–87.9)	73.5 (72.4–74.6)	8.1 (6.9–9.4)	99.3 (99.1–99.5)
NSICU	59.5	0.668 (0.608–0.729)	64.4 (52.3–75.3)	69.2 (64.0–74.1)	31.1 (23.8–39.2)	90.0 (85.7–93.4)
Neuro-ICU	55.5	0.812 (0.746–0.879)	93.8 (69.8–99.8)	68.7 (63.1–74.0)	14.0 (8.1–22.1)	99.5 (97.3–100)
SICU	67.5	0.701 (0.679–0.723)	66.2 (61.8–70.4)	74.0 (72.5–75.5)	26.6 (24.2–29.2)	93.9 (92.9–94.8)
Trauma SICU	63.5	0.727 (0.703–0.751)	72.3 (67.4–76.9)	73.1 (71.4–76.9)	25.2 (22.6–28.0)	95.5 (94.5–96.3)

AUROC, area under the receiver operating characteristic curve; CCU, cardiac care unit; CI, confidence interval; CVICU, cardiovascular intensive care unit; ICU, intensive care unit; MICU, medical intensive care unit; MICU/SICU, medical intensive care unit/surgical intensive care unit; NSICU, neurosurgical intensive care unit; Neuro-ICU, neurological intensive care unit; SICU, surgical intensive care unit; SOFA, Sequential Organ Failure Assessment.

Table S5. SAPS II score cutoffs for in-hospital mortality based on ICU type.

ICU type	Cutoff	AUROC (95% CI)	Sensitivity (95% CI)	Specificity (95% CI)	Positive predictive value (95% CI)	Negative predictive value (95% CI)
Overall	41.5	0.719 (0.711–0.727)	70.3 (68.8–71.8)	73.4 (72.9–74.0)	27.3 (26.4–28.2)	94.6 (94.3–94.9)
MICU	43.5	0.723 (0.708–0.737)	67.6 (64.8–70.3)	76.9 (75.8–78.1)	38.2 (36.1–40.4)	91.8 (91.0–92.6)
MICU/SICU	44.5	0.728 (0.712–0.744)	69.1 (66.0–72.0)	76.4 (75.2–77.6)	36.6 (34.3–38.9)	92.6 (91.8–93.4)
CCU	42.5	0.733 (0.710–0.755)	71.9 (67.5–76.0)	74.6 (72.7–76.4)	37.4 (34.2–40.8)	92.6 (91.3–93.8)
CVICU	41.5	0.739 (0.707–0.771)	74.6 (67.7–80.7)	73.1 (72.1–74.2)	7.3 (6.1–8.5)	99.0 (98.7–99.3)
NSICU	36.5	0.702 (0.646–0.757)	76.7 (65.4–85.8)	63.6 (58.2–68.7)	31.3 (24.6–38.6)	92.7 (88.5–95.7)
Neuro-ICU	38.5	0.732 (0.612–0.851)	68.8 (41.3–89.0)	77.6 (72.3–82.2)	14.3 (7.4–24.1)	97.9 (95.1–99.3)
SICU	37.5	0.687 (0.666–0.709)	71.7 (67.5–75.6)	65.8 (64.2–67.4)	23.0 (20.9–25.2)	94.2 (93.2–95.1)
Trauma SICU	39.5	0.740 (0.716–0.764)	72.9 (68.0–77.4)	75.2 (73.6–76.7)	26.9 (24.2–29.8)	95.7 (94.8–96.5)

AUROC, area under the receiver operating characteristic curve; CCU, cardiac care unit; CI, confidence interval; CVICU, cardiovascular intensive care unit; ICU, intensive care unit; MICU, medical intensive care unit; MICU/SICU, medical intensive care unit/surgical intensive care unit; NSICU, neurosurgical intensive care unit; Neuro-ICU, neurological intensive care unit; SICU, surgical intensive care unit; SOFA, Sequential Organ Failure Assessment.

Figure S1. Calibration plots of SOFA scores according to ICU type.

A. Total SOFA

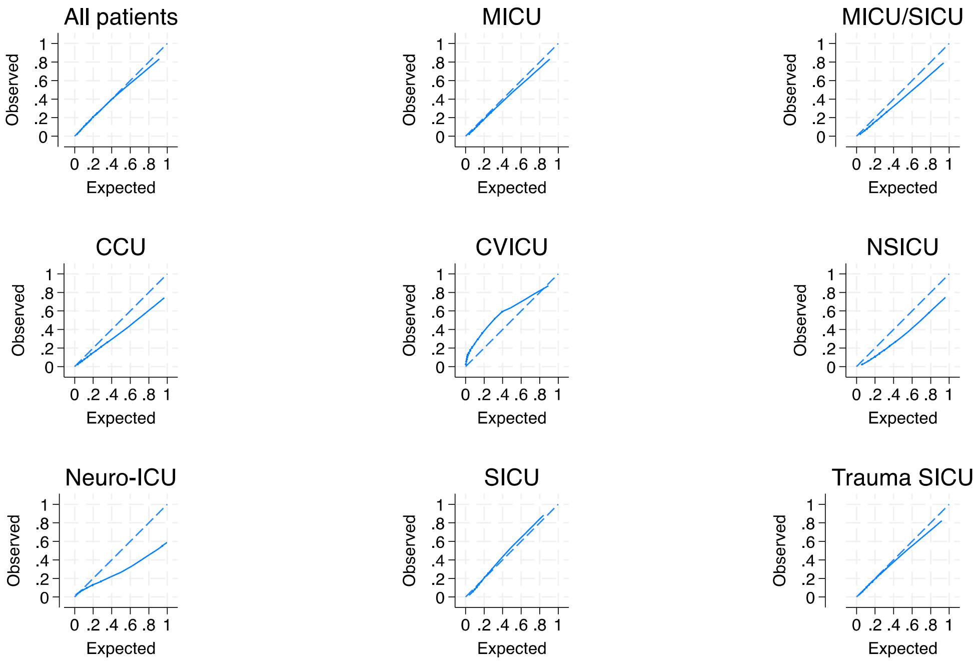

B. Cardiovascular SOFA

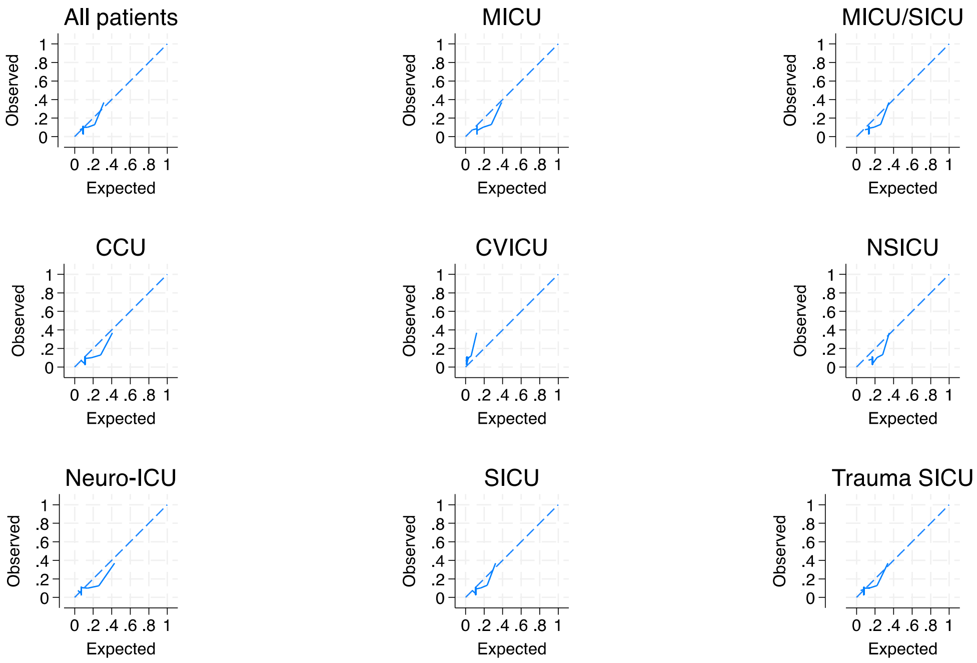

C. Coagulation SOFA

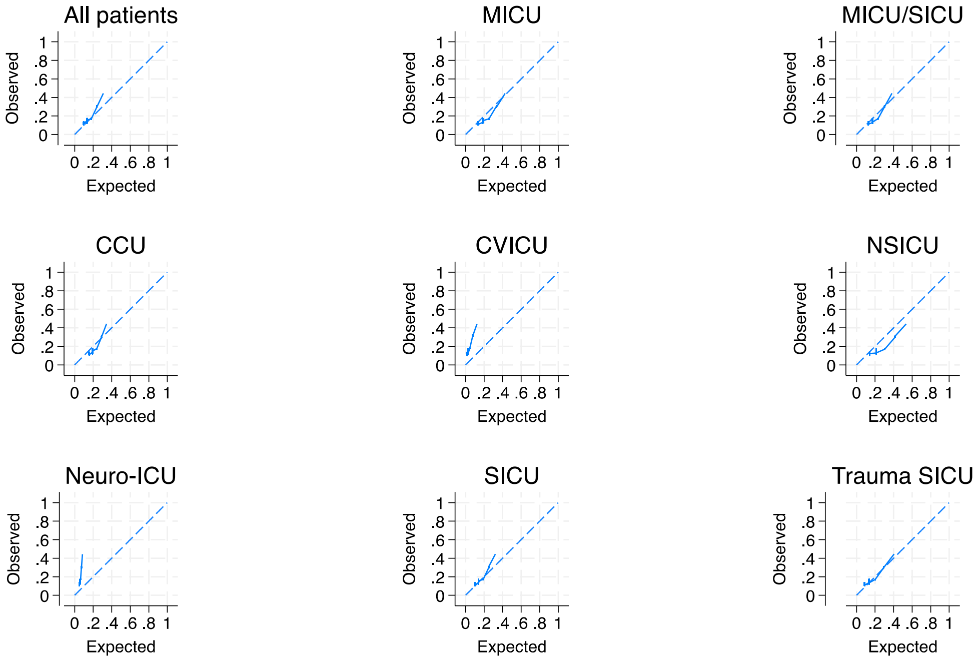

D. Respiratory SOFA

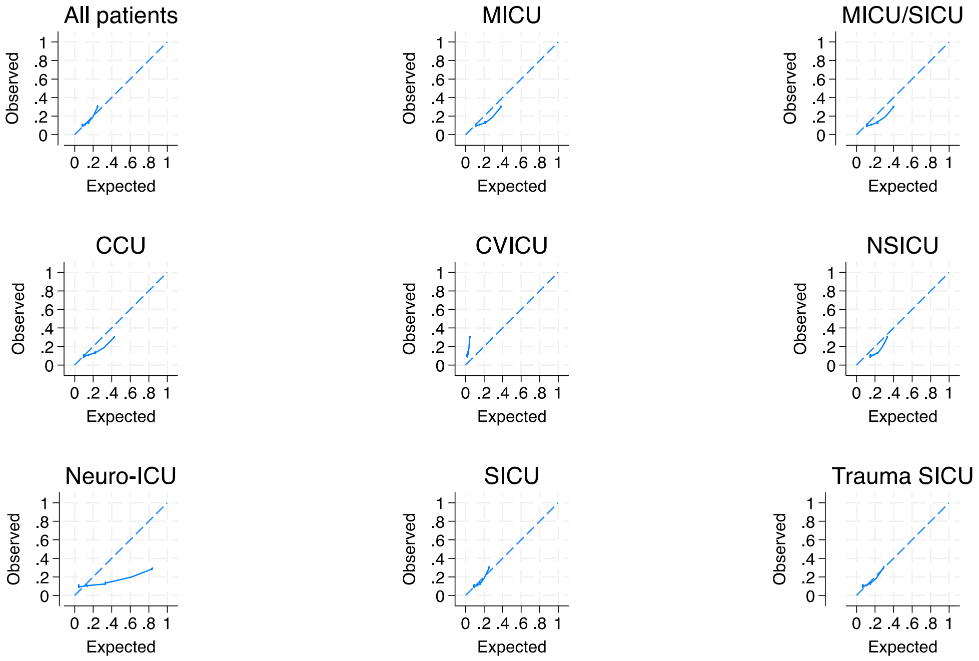

E. Hepatic SOFA

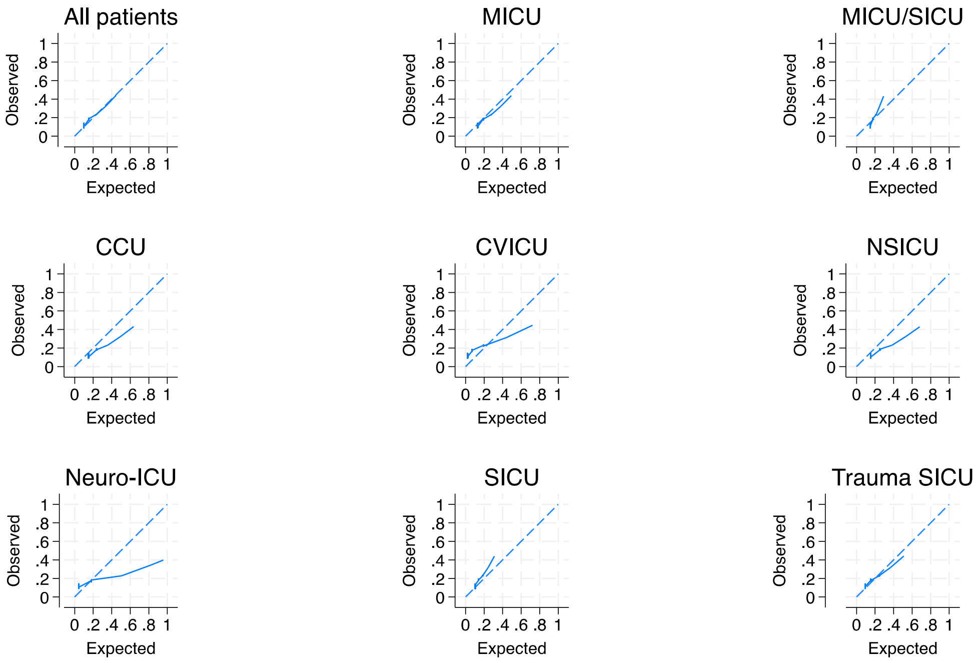

F. CNS SOFA

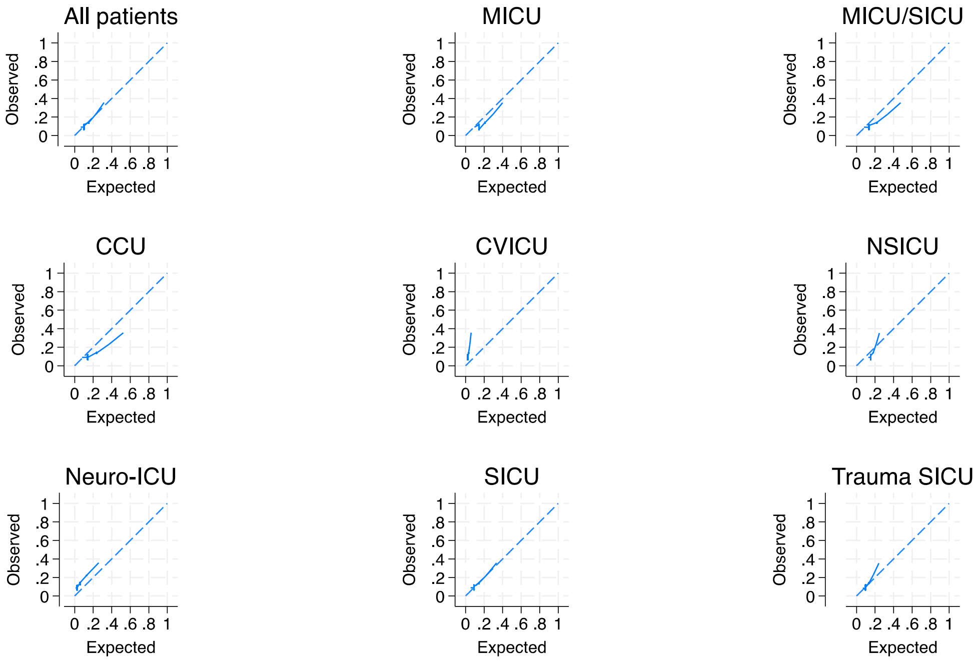

G. Renal SOFA

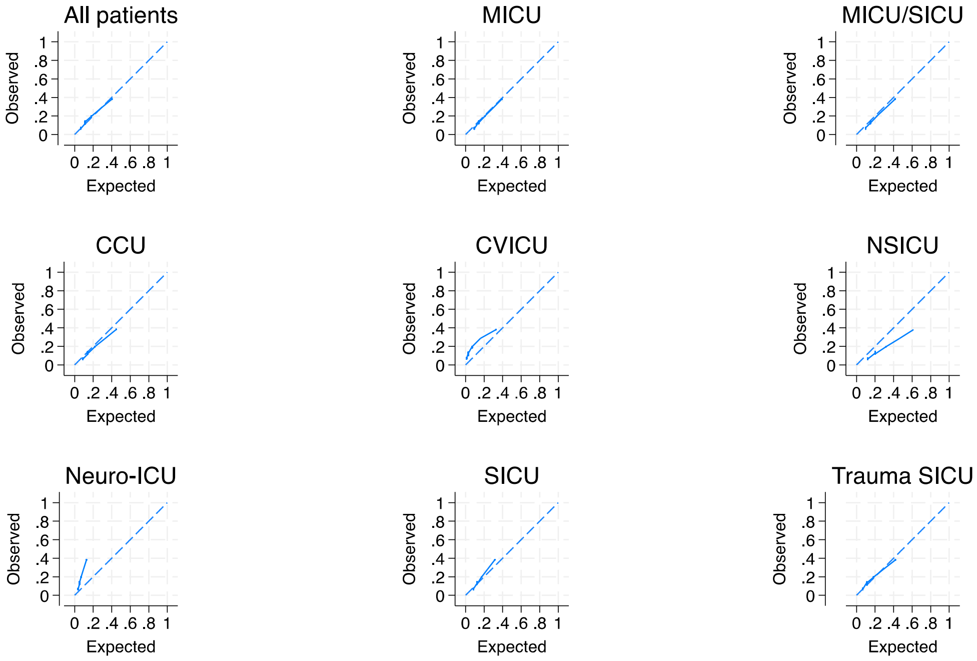
All patients
MICU
MICU/SICU
CCU
CVICU
NSICU
Neuro-ICU
SICU
Trauma SICU
Observed
Expected
0 .2 .4 .6 .8 1

Figure S2. Calibration plots of APACHE III scores according to ICU type.

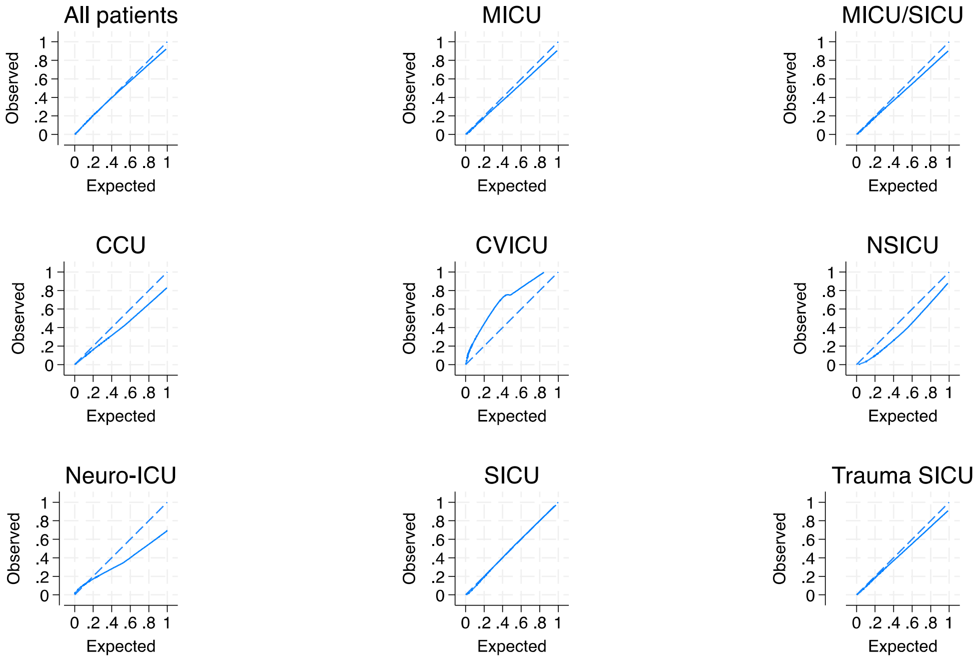

Figure S3. Calibration plots of SAPS II scores according to ICU type.

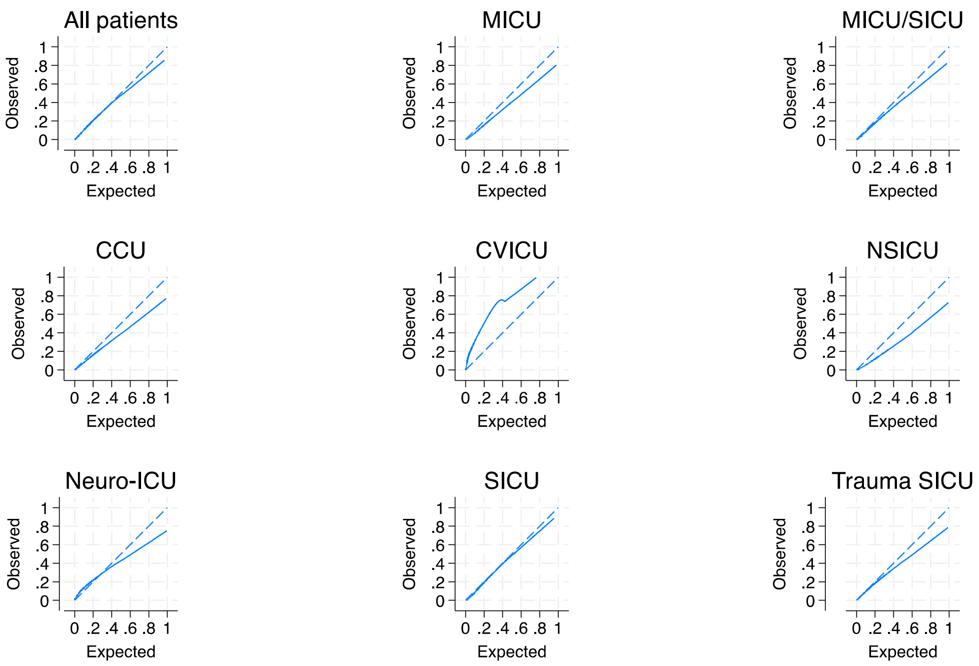

Supplement: Supplementary file 1 [file jcm-12-06402-s001.zip › jcm-2638407-supplementary.pdf]
